# Ankylosing Spondylitis: From Cells to Genes

**DOI:** 10.1155/2013/501653

**Published:** 2013-07-21

**Authors:** José Francisco Zambrano-Zaragoza, Juan Manuel Agraz-Cibrian, Christian González-Reyes, Ma. de Jesús Durán-Avelar, Norberto Vibanco-Pérez

**Affiliations:** Universidad Autónoma de Nayarit, Unidad Académica de Ciencias Químico Biológicas y Farmacéuticas, CP. 63190 Tepic Nay., Mexico

## Abstract

Ankylosing spondylitis (AS) is a chronic inflammatory disease of unknown etiology, though it is considered an autoimmune disease. HLA-B27 is the risk factor most often associated with AS, and although the mechanism of involvement is unclear, the subtypes and other features of the relationship between HLA-B27 and AS have been studied for years. Additionally, the key role of IL-17 and Th17 cells in autoimmunity and inflammation suggests that the latter and the cytokines involved in their generation could play a role in the pathogenesis of this disease. Recent studies have described the sources of IL-17 and IL-23, as well as the characterization of Th17 cells in autoimmune diseases. Other cells, such as NK and regulatory T cells, have been implicated in autoimmunity and have been evaluated to ascertain their possible role in AS. Moreover, several polymorphisms, mutations and deletions in the regulatory proteins, protein-coding regions, and promoter regions of different genes involved in immune responses have been discovered and evaluated for possible genetic linkages to AS. In this review, we analyze the features of HLA-B27 and the suggested mechanisms of its involvement in AS while also focusing on the characterization of the immune response and the identification of genes associated with AS.

## 1. Introduction

The spondyloarthropathies (SpA), now better denominated as spondyloarthritides (SpAs), are a diverse group of interrelated inflammatory arthritides that share multiple clinical features and common genetic predisposing factors. This group includes not only the prototypical disease, ankylosing spondylitis (AS), but also reactive arthritis (ReA), psoriatic arthritis (PsA), Crohn's disease, undifferentiated SpA, and juvenile-onset spondyloarthritis [1].

The clinical features of AS include inflammatory back pain, asymmetrical peripheral oligoarthritis, enthesitis, and specific organ involvement, such as anterior uveitis, psoriasis, and chronic inflammatory bowel disease [2]. Its major clinical features include sacroilitis, loss of spinal mobility, and spinal inflammation. Chronic inflammation leads to fibrosis and ossification, where bridging spurs of bone known as syndesmophytes form, especially at the edges of the inter-vertebral discs, producing the ankylosing [3].

AS affects men more often than women, at a ratio of 2 : 1 [4]. The prevalence of the disease is between 0.1 and 1.4% of general populations [2]. Studies conducted in different countries have shown that the incidence of AS varies from 0.5 to 14 per 100,000 people per year [2]. Diagnoses of AS are based more on clinical features than on laboratory tests; currently, diagnoses are made in accordance with the modified New York criteria (Table 1) [5].

AS is of unknown etiology but is considered an autoimmune disease that involves environmental and genetic factors. It is known to be highly heritable, as >90% of the risk of developing the disease has been shown to be genetically determined [6]. As in the case of most common heritable diseases, progress in identifying candidate genes associated with the disease, and their possible role in pathogenesis, is one of the challenges that must be confronted in the near future.

This review discusses recent advances in HLA-B27 studies, characterization of immune responses, and the identification of some genes associated with AS.

## 2. HLA-B27

Human leukocyte antigen (HLA)-B27 is a *Major Histocompatibility Complex* (MHC) Class I molecule that is encoded on chromosome 6p. It is ubiquitous among cell types and is highly expressed on antigen-presenting cells. After translation and tertiary folding, HLA-B27 heavy chains form heterotrimeric complexes with *β*2-microglobulin (*β*2m) and intracellular peptides derived from self-proteins, viruses, and bacteria. The association of HLA-B27 with AS was first described for *HLA* alleles and inflammatory diseases in 1973, and this association remains one of the best examples of a disease association with a hereditary marker [7, 8]; however, it does not explain the cause of the disease. Reports indicate that the risk of developing AS is approximately 5% in HLA-B27-positive subjects, but substantially higher for HLA-B27*-*positive relatives [9]. More than 90% of Caucasians with AS are HLA-B27-positive; however, most HLA-B27-positive individuals remain healthy, suggesting that other genes, both inside and outside the *MHC*, are involved in disease susceptibility [10–12]. Thus, *HLA-B27* may only account for perhaps 20 to 50% of overall genetic susceptibility to AS [13, 14].

Though there is no question that *HLA-B27* is the major susceptibility gene for AS, its mechanism of action remains unknown. There is strong evidence that different subtypes of HLA-B27 have distinct strengths of association with AS in specific populations. Some 100 HLA-B27 subtypes have been reported to date (http://hla.alleles.org/proteins/class1.html), but the number is increasing rapidly. Most of them differ from each other by only a few amino acids, but these changes are sufficient to alter the molecule's peptide-binding properties. HLA-B*2705 is present in all populations and appears to be the original or “parent” HLA-B27 molecule. Most of the other subtypes appear to have evolved along three pathways, defined by the pattern of amino acid substitutions in the first (*α*1) and second (*α*2) domains and through geographic patterns [15, 16]. The most common subtypes reported in association with AS are HLA-B*2705, B*2702, B*2704, and B*2707 [17–19]; whereas HLA-B*2706 and B*2709, which are common in Southeast Asia and Sardinia, have no association with AS, possibly due to amino acid differences in the B pocket of the HLA antigen-binding cleft that could modify the composition of the peptides that these HLA-B27 subtypes present [15, 20]. HLA-B*2706 and B*2709 differ from the AS-associated subtypes at residue 116 in the second domain. HLA-B*2706 differs from HLA-B*2704, which is highly disease-associated among the Chinese, in only two amino acid positions (H114D, D116Y), both of which reside on the floor of the F pocket of the peptide-binding groove. HLA-B*2709, meanwhile, differs from B*2705 by a single amino acid substitution at position 116 (D116H) [21, 22]. This position is a relevant polymorphism that gives rise to different repertoires of bound peptides and cytotoxic CD8^+^ T cells (CTL). As an example, pVIPR, a self-peptide derived from type I receptor of vasoactive intestinal peptide evokes autoreactive CTL responses in HLA-B*2705 individuals, mostly patients with AS, but not in HLA-B*2709 healthy individuals [23].

**Figure 1:**
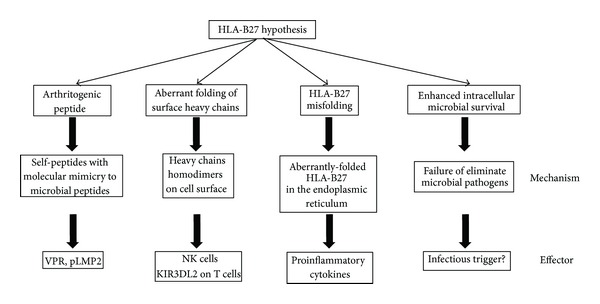
Proposed theories to explain the molecular pathogenic role of HLA-B27 in AS.

Several theories have been proposed to explain the molecular pathogenic role of HLA-B27 in AS, including the presentation of arthritogenic peptides, the aberrant folding of surface heavy chains, HLA-B27 misfolding, and enhanced intracellular microbial survival (Figure 1).

The dominant paradigm (arthritogenic peptide hypothesis) has been that self-peptides displayed by folded HLA-B27 become the target of autoreactive CD8^+^ T cells because they resemble microbial peptides, which does not occur with other HLA molecules. These T cells then cause cytotoxicity resulting in chronic inflammation. This hypothesis invokes the unique peptide-binding specificity of HLA-B27 as the problem. In support of this concept, HLA-B27-restricted CD8^+^ T-cell clones with specificity for bacteria or possibly self-peptides have been detected in both synovial fluid and peripheral blood of patients with ReA and AS [24]. Additionally, the finding of a self-peptide derived from the vasoactive intestinal peptide receptor (VIPR) that shows high sequence homology to an Epstein-Barr virus derived epitope of latent membrane protein 2 (pLMP2) peptide were reported in patients with AS. Although the VIPR peptide was chosen as a potential target that exhibit HLA-B27 subtype-dependent molecular mimicry with the EBV epitope, there was little cross-reactivity between VIPR and EBV-specific CD8^+^ T cells [25], but as yet there is no proof of the involvement of these peptides in the pathogenesis of AS.

The cell surface HLA-B27 homodimers hypothesis suggests that HLA-B27 heavy chain homodimers are produced on the cell surface during endosomal recycling [26]. The formation of disulphide bonds between the cysteine residue at position 67 (C_67_) in the B pockets of the peptide binding groove of two separate heavy chain molecules creates homodimers with no participation by *β*2m. HLA-B27 homodimers bind to specific receptors expressed on NK cells, T lymphocytes, and myelomonocytic cells; therefore, they could play a role in the pathogenesis of autoimmune disorders [27–29]. In support of this theory, it was found that HLA-B27 positive patients showed an increased number of NK cells and CD4^+^ T cells expressing KIR3DL2, a killer immunoglobulin-like receptor (KIR) that recognizes homodimers of HLA-B27 but not its heterodimers [29]. However, residue C_67_, which is critical for the formation of homodimers, exists in both HLA-B27 subtypes those that are related to AS and those that are not [30]. As counter arguments, no association has yet been reported between free heavy chains of HLA-B27 molecules and predisposition to AS [31], and the HLA-B2706 subtype, which is not related to AS, also forms homodimers [32].

The HLA-B27 misfolding hypothesis proposes that AS results from an accumulation of aberrantly-folded HLA-B27 in the endoplasmic reticulum (ER), that produces an inflammatory response [33]. ER stress resulting from the accumulation of misfolded heavy chains then activates the unfolded protein response (UPR), triggering a series of signaling pathways that culminate in the induction of ER-resident chaperones (BiP), which may induce cytokine production by macrophages, thereby promoting inflammation [34, 35]. Another pathway that can activate ER stress is the ER-overload response (EOR) to excessive membrane protein trafficking within the ER, which involves activation of the transcription of nuclear factor kappa B (NF-*κ*B) that can stimulate the synthesis of proinflammatory cytokines such as TNF-*α*, IL-1 and IL-6 in certain cell types [36].

The enhanced intracellular microbial survival hypothesis may also play a role in the pathogenesis of AS. This mechanism is based on the inability of HLA-B27-positive individuals to eliminate certain intracellular pathogens. Abnormal immune system activation or modulation can occur due to ineffective peptide loading into HLA-B27, leading to excessive viral or intracellular bacterial proliferation and delayed antigenic peptide clearance. Carriers of HLA-B27 are defective in the killing of intracellular bacterial species of the genera *Yersinia*, *Salmonella, Shigella,* and *Chlamydia* [37, 38], all of which—as is well documented—are involved in the triggering of reactive arthritis [39]; however, an infectious trigger for AS has yet to be demonstrated. 

It is highly likely that all of these mechanisms play some part in predisposing an individual to AS. Unfortunately, the precise role of HLA-B27 in pathogenesis remains unclear, but features that distinguish it from other genes and differences among its many subtypes have provided the basis for several putative explanations as to how it might predispose individuals to AS and mediate the disease.

## 3. Cells and AS

### 3.1. Th17 Cells

**Figure 2:**
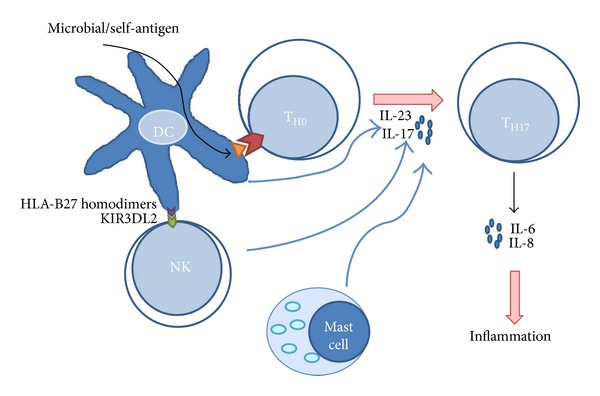
Possible role of Th17 in AS: dendritic cells (DC) could present an arthritogenic peptide derived from microbial pathogens or self-antigens to T_H0_ cells. The differentiation of these T cells could be influenced by IL-17 secreted by NK cells that recognize HLA-B27 homodimers and mast cells to induce the differentiation to T_H17_ cells that are involved in inflammation by molecules secreted, such as IL-6 and IL-8.

Interleukin 17 (IL-17) is a proinflammatory cytokine that contributes to the pathogenesis of several inflammatory diseases. One major source of IL-17 is a lineage of T cells known as T helper 17 cells (Th17 cells), but T cells, natural killer (NK) cells, mast cells, and neutrophils may also be involved [40]. It is well established that IL-17 activity contributes to various aspects of acute inflammation, because it mediates the release of IL-6 and IL-8 (Figure 2). The role of IL-17 in rheumatic diseases has been ascertained on the basis of findings that indicate that IL-17 promotes cartilage damage in a murine model [40].

It is also well known that IL-23 is able to induce IL-17 production and, therefore, is a crucial factor in the Th17 response [41]. In this regard, recent studies suggest that IL-23R could be one of the major genetic factors involved in susceptibility to AS [42]. Moreover, Th17 cells have been implicated in many experimental autoimmune diseases [41], and in the pathogenesis of several inflammatory diseases, including rheumatoid arthritis (RA) [43], psoriasis [44], and inflammatory bowel disease [45]. They also stimulate the formation of osteoclasts and, consequently, bone resorption and the recruitment of neutrophils and monocytes [46].

Several studies have been carried out in efforts to determine the role of different immune cells in the pathogenesis of AS, as well as CD4^+^ T cells producing IL-17 that have been associated with autoimmune diseases [47], particularly with inflammatory autoimmune diseases [48]. Mast cells infiltrated into synovial joints in SpAs have increased the expression of IL-17, which supports the notion that they could be a source of Th17 generation [49]. Moreover, Th17 cells have been shown to be involved in promoting the inflammatory process in AS [43]. Significantly elevated levels of Th17 cells have been reported in the peripheral blood of patients with AS [46, 50–52], suggesting that they could have a role in inflammation. Moreover, IL-17 and IL-23 have been found to be high as well in the serum of AS patients [42, 53, 54]. The role of Th17 cells in inflammation, and in AS, is supported by studies that have demonstrated that anti-TNF-*α* therapy reduces levels of IL-17 [51] and Th17 cells in patients with AS [46]. Though it is assumed that inflammation stimulates new bone formation, no concrete correlation between inflammation and osteoproliferation has yet been demonstrated [55]; moreover, inflammation and new bone formation can occur at distinct locations [56] and, apparently, anti-TNF-*α* therapy does not affect new bone formation in AS [57, 58]. Despite the fact that the relationship between inflammation and new bone formation remains controversial, there is a clear relationship between AS, Th17 cell levels, and the latter's cytokine secretion, which suggests an important role in the inflammatory process observed in AS (Figure 2).

### 3.2. Regulatory T Cells

Regulatory T cells (Tregs) mediate peripheral tolerance by actively suppressing effector T cells and inhibiting immune-mediated tissue damage. Tregs were first identified by the expression of CD25, but now they are characterized by the expression of the intracellular transcription factor, FoxP3 [59]. Tregs function by maintaining immune tolerance and preventing inflammatory diseases. In addition, they have been implicated in the regulation of almost every adaptive immune response and, therefore, also in inflammatory responses, by using appropriated mechanisms that inhibit targeted cell populations [60].

In the case of AS, few studies have been carried out to analyze the levels of Tregs in the peripheral blood of patients; however, low percentages of Treg cells have been reported in the peripheral blood [46, 51, 69, 70], and in the synovial fluid [71] of patients with AS, suggesting an imbalance between Tregs and the adaptive immune response. Moreover, AS patients treated with anti-TNF therapy showed similar levels of Treg cells to those observed in healthy subjects [46]. These data suggest a possible role of Tregs in AS, and Th17/Tregs imbalance has been proposed as playing a novel role in AS [69].

### 3.3. NK Cells

The recognition that both the adaptive and innate immune responses play key roles in AS led us to focus on NK cells as a target for improving our understanding of the pathogenesis of AS. NK cells are major components of innate immunity and provide surveillance during early defense against virus, intracellular bacteria, and cancer cells [72], but they have also been associated with autoimmunity. NK cells can be identified by the expression of CD56 and the lack of the CD3 complex [73]. Decreased numbers and impaired function of peripheral blood NK cells in patients with autoimmune diseases such as systemic lupus erythematosus (SLE), multiple sclerosis, diabetes, RA, and psoriasis have been reported [74–78]. However, the frequencies of circulating CD3^−^CD56^+^ NK cells have been reported to be higher in AS patients [73, 79], as have those of the CD56^dim⁡^CD16^+^ subsets, but not CD56^bright^CD16^+^ [80]. The role of NK cells in AS has been supported by the finding that the HLA-B27 protein is specifically recognized by the NK-inhibitory receptor KIR3DL1 [73]. The killing activity of NK cells is balanced by the signals transduced by both inhibitory and activating receptors [73]. Surface receptors of NK cells have been evaluated in several studies, and NKp44^+^ and NKp46^+^ receptors have been evaluated in patients with AS, while NKp44 expression has been found to be elevated in the ileum of patients with AS. Also, these cells secreted increased amounts of IL-22 [73, 81]. Indeed, healthy HLA-B27-positive subjects have similar NK cell levels to those in AS patients. Increasing evidence points to a role of the KIRs in the development of autoimmune diseases. In particular, a positive association of KIR3DS1 (an activating receptor) and a negative association of KIR3DL1 (an inhibitory receptor) with AS have been reported [82]. Additionally, it is known that KIR3DL2 binds to free H chain forms of HLA-B27 [83]. Genetic polymorphisms of KIRs genes have been studied by some groups, finding that KIR2DL1, KIR2DL5, KIR2DS5, KIR3DS1, and KIR3DL1 are all associated with AS, though in different populations [82, 84–91]. These data suggest that NK cells could play a relevant pathogenic role in AS via the expression of KIRs [92].

## 4. Molecules, Their Genes and AS

### 4.1. CCR6

The chemokine (C-C motif) receptor 6 (CCR6) is expressed on B cells, a fraction of T cells, and immature DCs, and studies have shown that it is a specific marker for Th17 cells that distinguishes them from other helper T cells. Moreover, CCR6 has been shown to be important for B-lineage maturation and antigen-driven B-cell differentiation and may regulate the migration and recruitment of dendritic cells (DCs) and T cells during inflammatory and immunological responses. CCR6^+^ human memory T cells have a low stimulation threshold for IL-10 production and, consequently, secrete IL-10 after suboptimal stimulation by autologous DCs [93]. 

CCR6 is considered an important receptor that guides effector T cells into inflamed tissue, thus favoring the Th17 phenotype and downregulating the Tregs. Thus, CCR6^+^ T cells play a central role in balancing regulatory and inflammatory processes during homeostasis and inflammation [94]. It has also been reported that CCR6^−^ deficient mice have altered CD4^+^ T-cell responses, including reduced hypersensitivity and enhanced delayed type hypersensitivity responses [95], all of which supports the role of CCR6 in homeostasis.

CCR6 are also involved in several autoimmune diseases, including psoriasis and RA [96–98]; however, *CCR6* polymorphisms have not been associated with AS in the few populations that have been analyzed [98, 99], despite the fact that some studies have demonstrated that it is expressed on the Th17 cells of AS patients [100, 101] and that these patients have a higher proportion of these cells [43].

### 4.2. Negative Costimulatory Molecules: CTLA-4 and PD-1

Cytotoxic T-lymphocyte antigen 4 (CTLA-4, CD154) is a costimulatory molecule that is expressed by activated T cells and interacts with the B7 molecules on the surface of antigen-presenting cells to induce downregulation of T-cell activation. CTLA-4, which is encoded by the *CTLA4* gene located on chromosome 2p33, is a structural homologue of CD28 [61]. Engagement of CTLA-4 appears to regulate ongoing T-cell responses and induce peripheral T-cell tolerance, while the absence of this function appears to be involved in autoimmunity [102]. In addition, CTLA-4 is highly expressed by regulatory T cells and could play an important role in their functioning [103]. Theoretically, polymorphisms of *CTLA4* that reduce CTLA-4 expression may cause autoimmune T-cell clonal proliferation, thus contributing to the pathogenesis of autoimmune diseases [61]. The *CTLA4* gene has many single nucleotide polymorphisms (SNP), some of which are present in regulatory positions, while others appear in 3′ UTR, but the most important one is the leader sequence (+49 A/G; rs231775) [62, 104, 105]. The +49-A/G is located at position +49 of the first exon of the *CTLA4* gene, where it provokes a threonine-to-alanine change in amino acid 17 of the leader peptide [103]. It has been reported that +49 A/G polymorphism in *CTLA4* gene alters the intracellular distribution of CTLA-4, IL-2 production, and T-cell proliferation [103, 106], suggesting their possible role in autoimmune diseases. This SNP have been analyzed in patients with AS in different populations (Table 2), but so far results are negative [61–63], though higher levels of circulating CTLA-4 in SpAs [107] and association of the +49-GG genotype with circulatory C-reactive protein in patients with AS [63] have been found, indicating a possible role in the pathogenesis of AS.

The programmed cell death-1 (*PDCD1*, also known as *PD1*) gene is one of the costimulatory genes located on chromosome 2q37.3. It encodes the surface receptor PD-1, an inhibitory immunoreceptor expressed on activated T cells, B cells, and myeloid cells belonging to the immunoglobulin superfamily B7-CD28 [108]. PD-1 is expressed in a variety of hematopoietic cells on the periphery after stimulation by antigen receptor signaling and cytokine receptors. Two PD-1 ligands have been described (PD-L1 and PD-L2), and their expression is regulated by the inflammatory environment, cytokines such as TNF-*α*, type 1 and 2 interferons, IL-2, IL-7, and IL-15 [59].

The co-stimulatory pathways consisting of the PD-1 receptor and its ligands deliver inhibitory signals that regulate the balance among T-cell activation, tolerance, and immune-mediated tissue damage [59]. Various SNPs in the *PD1* gene have been identified, such as *PD1.1* (rs36084323), *PD1.3* (rs11568821), *PD1.5* (rs2227981), and *PD1.9* (rs2227982). Among these, *PD1.3*, *PD1.5*, and *PD1.9* have been associated with autoimmune disorders in different ethnic groups [134]. In the case of AS, despite controversies among studies of the polymorphism associated with the disease, it appears that *PD1.3, PD1.5,* and* PD1.9* are all candidates for association (Table 2) [64–68]. However, it is necessary to study these polymorphisms or conduct meta-analysis in additional populations, before these associations can be confirmed.

### 4.3. Endoplasmic Reticulum Aminopeptidase 1 (ERAP1)

*ERAP1* is the term currently accepted by the human genome organization (HUGO) nomenclature committee (HGNC), though in the past it was known by such names as endoplasmic reticulum aminopeptidase associated with antigen processing (ERAAP), adipocyte-derived leucine aminopeptidase (A-LAP), and aminopeptidase regulating tumor necrosis factor receptor I (TNFRI) shedding (ARTS-1). ERAP1 is a zinc aminopeptidase belonging to the M1 family of the metallopeptidases that share the consensus GAMEN and HEXXH(X)18E zinc-binding motifs [135]. Two major ERAP1 protein isoforms are generated: the longer isoform, a (ERAP1-*a*) and the shorter isoform, b (ERAP1-*b*). It has been reported that the isoform ERAP1-*b* is more abundant than ERAP1-*a* [136]. Because ERAP1 is highly polymorphic multiple splice variants with potential effects on biological functions have been described, for example, rs2287987 (M349V) located on the active site, rs17482078 (R725Q), and rs27044 (Q730E), which are exposed on the inner surface of the C-terminal cavity and could affect the substrate sequence or length specificity. Other polymorphisms, such as rs26653 (R127P), rs30187 (K528R), and rs10050860 (D575N), localized at domain junctions, reduce either specificity or aminopeptidase activity toward a synthetic peptide substrate by altering the conformational change between open and closed conformations [137]. 

The association of *ERAP1* SNPs with AS can be explained from a functional perspective. The protein ERAP1 has three known biological functions. First, in the endoplasmic reticulum, ERAP1 acts as a molecular ruler, trimming peptide antigens to optimal length for binding to MHC class I molecules [138]. Complex proteins are initially degraded in the cytosol by the proteasome complex to generate peptide fragments up to 25 amino acids in length [139]. These antigenic peptides, and their N-terminal extended precursors, are subsequently transported into the ER by the transporter associated with antigen processing (TAP) that preferentially transports peptides of 8–16 residues in length [140–142]. Nascent MHC class I molecules typically bind short peptide fragments 8-9 residues long and transport them to the cell surface for presentation to T cells. ERAP1 is expressed in the lumen of the ER, where peptide loading to MHC class I molecules takes place. Here, ERAP1 preferentially trims substrates 10–16 residues in length; whereas peptides 8-9 residues in length are optimal for binding MHC class I molecules [143, 144]. Second, the cleavage of cell surface receptors by proinflammatory cytokines such as TNFR1 [145], IL-1R2 [146], and IL-6R*α* [147] results in the downregulation of their intracellular signaling. For this reason, the malfunctioning of ERAP1 would lead to either an increase or decrease in the number of cell surface receptors available for these cytokines, thus propitiating proinflammatory effects and, finally, raising disease susceptibility to AS, though some polymorphisms of ERAP1 associated with AS do not influence the cytokine receptor levels in patients with this disease [148]. Third, ERAP1 is involved in the activation of macrophages induced by lipopolysaccharide (LPS) and interferon (IFN)-*γ* [149]. 

The first confirmed association of *ERAP1* with AS was reported by the Wellcome Trust Case-Control Consortium and Australo-Anglo-American Spondyloarthritis Consortium (WTCCC/TASC) in 2007. They used 14,500 nonsynonymous SNPs to discover the *ERAP1* association in AS. This was the first non-*MHC* gene for which a definitive AS-association was observed [109]. In that study, the minor allele frequency of the *ERAP1* SNP rs30187 and rs27044 in AS patients was considerably higher than in controls, and those variants have been repeatedly confirmed by nearly all population studies as conferring strong susceptibility to this disease. At the same time, this study indicates that in the *MHC* locus *HLA-B27* confers the greatest risk of AS susceptibility, with an attributable risk of 50%; *ERAP1* is the second strongest association, with an attributable risk of 26% [109]. Recent genome-wide association studies (GWAS) have revealed numerous *ERAP1* polymorphisms that are associated with AS and strongly related to the *HLA-B27 MHC* I allele [110, 150]. One GWAS study found that the polymorphisms of *ERAP1* only affect AS risk in HLA-B27-positive individuals [111]. The association of AS with *ERAP1* has been replicated in multiple cohorts and ethnicities, including a family-based association study [112], case-control studies [113–123], and meta-analyses [124, 125] of Canadian, British, Portuguese, Chinese, Hungarian, Korean, Spanish, and Iranian populations. Those studies were able to replicate the association of the rs30187 and rs27044 SNP in all the aforementioned populations, with the sole exception of Pazar's study, where the SNP rs30187 failed to show any significant connection with AS susceptibility [117]. It is important to mention that the North American Spondylitis Consortium (NASC) studied multiplex AS families, finding and reporting, for the first time, a novel haplotype *ERAP2* associated with AS [112]. In several disease models, the association of *ERAP1* with AS in HLA-B27-positive cases is consistent. There, aberrant trimming of peptides or the presentation of ERAP1 and HLA-B27 are involved in the pathogenesis of HLA-B27-associated AS. These findings suggest that ERAP1 participates in AS pathogenesis with associated alleles that reduce the risk of disease through a mechanism that involves altered peptide presentation by MHC class I, though a great deal of additional experimental research is necessary to validate this. In Table 3, studies of association of *ERAP1* and AS are summarized.

### 4.4. Receptor for the Fc Fragment of IgG (Fc*γ*R)

Receptors for the Fc fragment of IgG (Fc*γ*R) form a group of type I transmembrane glycoproteins belonging to the Ig superfamily and expressed mostly on leucocytes, providing a critical link between the humoral and cellular arms of the immune response. Fc*γ*R has three major functions: (1) positive and negative regulation of cell activation; (2) Ig transport and regulation of Ig homeostasis; and (3) uptake of the immune complex (IC) for the degradation and promotion of antigenic peptides for antigen presentation [151, 152] that can trigger effector mechanisms, such as antibody-dependent cellular cytotoxicity (ADCC), phagocytosis, degranulation, and cytokine production via immune tyrosine activating or inhibitory motifs (ITAM or ITIM). Three classes of leukocyte Fc*γ*R are currently distinguished: Fc*γ*RI (CD64), Fc*γ*RII (CD32), and Fc*γ*RIII (CD16). Fc*γ*RI is a high-affinity receptor that binds both monomeric IgG and immune complexes; whereas the affinity of IgG is low for Fc*γ*RII and intermediate for Fc*γ*RIII [153]. Fc*γ*RIa, Fc*γ*RIIa, Fc*γ*RIIc, and Fc*γ*RIIIa are activating receptors characterized by the presence of an immunoreceptor tyrosine-based activation motif (ITAM); Fc*γ*RIIIb is unique, as it is anchored by a glycosylphosphatidylinositol (GPI) anchor. In contrast, Fc*γ*RIIb is an inhibitory receptor that contains an immunoreceptor tyrosine-based inhibitory motif (ITIM) in its cytoplasmic domain. The inhibitory receptor Fc*γ*RIIb plays a major role in controlling the antibody and immune response and the development of autoimmune diseases [154–156]. Fc*γ*RIIB includes two isoforms, Fc*γ*RIIB1 and Fc*γ*RIIB2, which transduce inhibitory signals that downregulate immune functions triggered by activating receptors. For instance, Fc*γ*RIIB engagement triggers the blockade of BCR-induced B-cell activation, once it joins with BCR [157]. The opposed signaling pathways of activating and inhibitory Fc*γ*R act in concert to determine the magnitude of the effector cell responses in immune-complex inflammation and autoimmune disease. In noninflamed tissues, the ratio of activating to inhibitory Fc*γ*R is low, but it increases markedly in an inflamed environment. Therefore, activating Fc*γ*R promotes disease development, while inhibitory Fc*γ*R contributes to protection in two different ways: first, through the downregulation of effector cell responses and, second, by maintaining peripheral tolerance [158].

Some studies have found promoter mutations that induce lower *Fc*γ*RIIB* expression levels in individuals that are susceptible to autoimmune diseases [159, 160]. Likewise, it has been reported that Fc*γ*Rs may play a role in the pathogenesis of RA and SLE. Recently, based on specific locus genetic loci studies, *Fc*γ*RIIB* was reported to be associated with AS development in a case-control study in Han Chinese. That study indicated that rs10917661 may be a novel SNP involved in AS genetic predisposition [161]. In a previous study, we reported the association of rs1801274 (H131R) of *Fc*γ*RIIA* and rs396991 (V158F) of *Fc*γ*RIIIA* with AS in a small group of patients, having found that the *Fc*γ*RIIA-HH* and *Fc*γ*RIIIA-VV* variants are associated with AS [162]. We therefore suggested that these polymorphisms could be related to the IgG3 immune response against bacterial antigens, as previously reported [163], and to human and bacterial HSP60 [164]. Moreover, a relation between the *Fc*γ*RIIIA-VV* genotype and the response to infliximab has also been found [165]. These discoveries are important for our understanding of the association between FcR*γ* and the pathogenesis of AS, but more research is required in this field, including replication of the association of *Fc*γ*RIIB*, *Fc*γ*RIIA*, and *Fc*γ*RIIIA* with AS in multiple cohorts and ethnicities, to ascertain whether these SNPs are linked with predisposition to AS.

### 4.5. Tumor Necrosis Factor-Alpha (TNF-*α*)

Tumor necrosis factor-alpha is a highly potent proinflammatory molecule and a key signaling component of the immune system that is strongly induced after infection or tissue injury [166]. This cytokine is known to be present at higher concentrations in patients with AS, RA, and PsA. The important role of TNF in these diseases has been proven by their successful treatment with anti-TNF drugs [166–168], particularly AS patients respond well to TNF-*α* antagonist therapy [169].

Indeed, anti-TNF-*α* therapy has become the standard of care for AS patients over the last decade. Several studies have shown that TNF-*α* blocking agents, such as infliximab, etanercept, and adalimumab are highly efficacious in controlling inflammation and improving the clinical assessment of AS patients [167, 170–172].

A prospective study showed that three years of TNF-*α* blocking therapy result in a significant increase in the bone formation marker after three months, a result that continued at a higher level up to three years, leading to a bone turnover balance that favors bone formation, in combination with continuous improvements in bone mineral density (BMD) in the lumbar spine [173]. Furthermore, bisphosphonates in conjunction with anti-TNF agents have a synergistic effect that provides additional increases in the BMD of AS patients [174]. This suggests that TNF-*α* blockades may even reinforce extensive bone formation by suppressing the inflammatory component of AS. 

The *TNFA* gene contains several SNPs [175], the most widely studied of which is −308 A/G SNP (rs1800629), and −238 A/G (rs361525) in the promoter region. It has been found to be involved in many diseases due to its ability to modify cytokine levels and clinical outcomes [176, 177]. The −308 A/G and −238 A/G transition seems to be associated with susceptibility to autoimmune diseases, although other SNP at −1031 (rs1799964), −863 (rs1800630), −857 (rs1799724), and −238 (rs361525) in the promoter region of *TNFA* have been evaluated in AS (Table 4).

However, there are contradictory results, because some studies did not found association between the −238 and −308 polymorphisms of *TNFA* with AS [126–131], although the A allele and the AA genotype of *TNFA* (−308 and −238) have been associated with AS, and with a higher production of TNF-*α* in two populations, which could leads to a state of latent inflammation and subsequent tissue damage, confirming the participation of elevated levels of this cytokine in establishing the inflammatory process in this disease [132, 133]. In this regard, a Swedish group has reported that patients with AS, AR, and PsA who carry the GG genotype exclusively showed a good response to anti-TNF-*α* therapy, whereas a moderate response was associated with the −308 A/G genotype and unresponsiveness with the AA genotype [178]. These results emphasize the importance of *TNFA* polymorphism as a predictor of responses to TNF-*α* blocking agents.

In contrast, a meta-analysis conducted by Lee and Song (2009) failed to demonstrate any association of the *TNFA* −308 polymorphisms with AS in Europeans [127], leaving several open questions as to the importance of the role of *TNFA* polymorphisms in AS. 

TNF-*α* is synthesized and expressed on the cell surface as a transmembrane protein (tmTNF-*α*) that can be processed by the TNF-*α* converting enzyme to generate a soluble form (sTNF-*α*) [179]. Both the soluble and transmembrane forms of TNF-*α* are biologically active in their trimeric forms [180, 181] and act by binding two different receptors, TNFR1, which is activated by both sTNF-*α* and tmTNF-*α*, and TNFR2, which is activated mainly by tmTNF-*α* [167, 182]. 

TNFR-1 is the primary signaling receptor on most cell types and accounts for the majority of the proinflammatory, cytotoxic, and apoptotic effects classically attributed to TNF-*α* [183, 184]. In contrast, TNFR2 predominantly mediates signals promoting lymphocyte activation and proliferation [181]. Therefore, at least in the inflammatory environment, the TNF-*α*/TNFR2 pathway is critical for stabilizing the Treg-cell pool that is required to restrain the magnitude and length of an inflammatory immune response and prevent damage to self-tissues [185, 186]. Moreover, several polymorphisms of the TNFRs may contribute to the development of an abnormal immune response in AS.

In the Mexican population, the work group of Corona-Sanchez et al. found a high frequency of the AA genotype of the −383 *TNFR1* polymorphism in patients with AS. In addition, the A allele is significantly associated with a higher risk of AS [187].

### 4.6. Concluding Remarks

Ankylosing spondylitis is a multifactorial disease. HLA-B27 has been associated with AS since 1973; however, different pathways have been described to explain this association. These pathways include the KIRs receptors that could interact with HLA-B27. These receptors are expressed by NK cells, but the involvement of NK cells in AS takes place not only through recognition of HLA-B27, but also through the secretion of proinflammatory cytokines and their effect on Tregs. New participants in the inflammatory process are Th17 cells that induce the secretion of cytokines, such as TNF-*α*, which is clearly involved in the pathogenesis of AS.

The genetic factor has been analyzed in studies of several of the genes involved in the immune response, and particularly in inflammatory responses. The SNPs included suggest that these variations could play a role in AS because of their functional effect on the expression of the genes. Describing the cells and genes associated with this disease is the first step towards the eventual detection of possible therapeutic targets that could be used to improve current treatments and patients' quality of life. However, it is necessary to analyze the functional role of these genes and cells in the pathogenesis of the disease in order to reach an understanding of the mechanisms of these cells and genes in the pathogenesis of AS.

## Figures and Tables

**Table 1:** Modified New York criteria 1984 for ankylosing spondylitis [5].

*Clinical criteria *	
(i) Low back pain and stiffness for longer than 3 months, which improve with exercise but are not relieved by rest.	
(ii) Limitation of motion of the lumbar spine in both the sagittal and frontal planes.	
(iii) Limitation of chest expansion relative to normal values correlated for age and sex.	

*Radiological criterion *	
Sacroilitis grade ≥ 2 bilateral, or grade 3-4 unilateral.	

For definite ankylosing spondylitis the radiological criterion and at least one clinical criterion must be satisfied.

**Table 2:** Association studies between *CTLA4* +49-A/G and *PD1* polymorphisms with ankylosing spondylitis.

Gene	Population	Year	Study type	SNP	Association	Reference
*CTLA4 *	Iranian	2010	Case control	+49-A/G (rs2317754)	NS	[61]
	European	2001	Case control		NS	[62]
	Taiwanese	2010	Case control		NS	[63]

*PD1*	Han Chinese	2009	Case control	*PD1.3*, *PD1.5*, *PD1.9 *	NS NS Risk	[64]
	Iranian	2011	Case control	*PD1.3*, *PD1.9 *	NS NS	[65]
	Taiwanese	2011	Case control	*PD1.1*	Risk	[66]
	Korean	2006	Case control	*PD1.5*, *PD1.9 *	NS Risk	[67]
	Chinese	2011	Case control	*PD1.9*	Risk	[68]

*PD1.1* (rs36084323), *PD1.3* (rs11568821), *PD1.5* (rs2227981), and *PD1.9* (rs2227982).

NS: not significant.

**Table 3:** Association studies for *ERAP1* and ankylosing spondylitis.

Population	Year	Study type	SNP	Association	Reference
UK	2007	GWAS	rs30187 rs27044 rs17482078 rs10050860 rs2287987	Risk Risk NS NS NS	[109]

Australian, British, US^1^	2010	GWAS	rs27434 rs27037	Risk Risk	[110]

UK, Australian, Canadian	2011	GWAS	rs30187	Risk	[111]

Canadian	2010	GWAS	rs30187 rs27044 rs10050860	Risk No association No association	[112]

Portuguese	2009	Case control	rs27044, rs17482078 rs10050860 rs30187 rs2287987	Risk NS NS Risk NS	[113]

UK^1^	2009	Case control	rs28366066 rs26653 rs2287987 rs27434 rs30187 rs10050860 rs469783 rs17482078 rs1065407 rs13167972	Protection Risk Protection Risk Risk Protection Risk Protection Protection Protection	[114]

Canadian	2009	Case control	rs27044 rs10050860 rs30187 rs26618 rs26653 rs3734016	NS Protection Risk NS Risk NS	[115]

Han Chinese^1^	2009	Case control	rs27037 rs27980 rs27433 rs27038	Risk Protection Risk Risk	[116]

Hungarian	2010	Case control	rs27044 rs17482078 rs10050860 rs30187 rs2287987	Risk NS Protection NS Protection	[117]

Korean	2010	Case control	rs27044 rs30187 rs17482078 rs10050860 rs2287987	Risk Risk No association No association No association	[118]

Han Chinese	2011	Case control	rs27038 rs27037	Risk Risk	[119]

Han Chinese	2011	Case control	rs27044	NS	[120]

Spanish	2011	Case control	rs17481856 rs17482078 rs30187 rs2287987 rs27895 rs27044 rs26653 rs10050860	NS Risk Risk Risk NS NS Risk Risk	[121]
Han Chinese	2011	Case control	rs27434, rs27529	Risk Risk	[122]

Iranian	2012	Case control	rs30187 rs27434 rs469876 rs13167972	Risk Risk NS NS	[123]

European, Asian	2011	Meta-analysis	rs27044 rs30187	Risk Risk	[124]
European	2011	Meta-analysis	rs17482078, rs10050860 rs2287987	Protection Protection Protection	

All cases	2012	Meta-analysis	rs27044 rs17482078 rs10050860 rs30187 rs2287987 rs27037	Risk Risk Protection Risk Protection Risk	[125]

GWAS: genome-wide association study.

^1^Only the SNPs associated with AS were included.

NS: not significant.

**Table 4:** Association studies of *TNFA* polymorphisms with ankylosing spondylitis.

Population	Year	Study type	SNP	Association	Reference
Greek	2009	Case control	rs1799724 rs1800629 rs361525	Risk NS Not H-W* equilibrium	[126]

Various	2011	Meta-analysis	rs361525 rs1800629	NS NS	[127]

Various	2010	Meta-analysis	rs1800629 rs361525	NS NS	[128]

German	2011	Case control	rs1800629 rs361525	NS NS	[129]

Colombian	2012	Case control	rs1800629	NS	[130]

Mexican	2006	Case control	rs1800629 rs361525	NS NS	[131]

Iranian	2009	Case control	rs1800629 rs361525	NS Risk/Protection (allele A/G)	[132]

Taiwanese	2007	Case control	rs1800629 rs361525	NS protection	[133]

*H-W: Hardy-Weinberg.

NS: not significant.
